# Chronic High Dose Zinc Supplementation Induces Visceral Adipose Tissue Hypertrophy without Altering Body Weight in Mice

**DOI:** 10.3390/nu9101138

**Published:** 2017-10-18

**Authors:** Xiaohua Huang, Dandan Jiang, Yingguo Zhu, Zhengfeng Fang, Lianqiang Che, Yan Lin, Shengyu Xu, Jian Li, Chao Huang, Yuanfeng Zou, Lixia Li, De Wu, Bin Feng

**Affiliations:** 1 Animal Nutrition Institute, Sichuan Agricultural University, Chengdu 611130, China; hxh3028@163.com (X.H.); dandanjiang17@163.com (D.J.); yingguozhu@outlook.com (Y.Z.); zfang@sicau.edu.cn (Z.F.); clianqiang@hotmail.com (L.C.); able588@163.com (Y.L.); shengyu_x@hotmail.com (S.X.); lijian522@hotmail.com (J.L.); 2 Key Laboratory of Animal Disease-Resistant Nutrition of Ministry of Education, Sichuan Agricultural University, Chengdu 611130, China; 3 College of Veterinary Medicine, Sichuan Agricultural University, Chengdu 611130, China; huangchao@sicau.edu.cn (C.H.); yuanfengzou@sicau.edu.cn (Y.Z.); lilixia905@163.com (L.L.)

**Keywords:** zinc, visceral adipose tissue, hypertrophy, glucose clearance, leptin, IL6, AKT

## Abstract

The trace element zinc plays an important role in human life. Zinc deficiency impairs growth, reproduction, metabolism and immunity in both human and animals. Thus, zinc supplementation is recommended in daily life. However, the effect of long-term chronic zinc supplementation on adipose homeostasis has not been well elucidated. In the current study, mice were supplemented with zinc sulfate in the drinking water for 20 weeks. The results suggested that chronic zinc supplementation impaired systemic glucose clearance after exogenous insulin or glucose challenges, as compared to the control mice. Further study revealed that chronic zinc supplementation made no difference to body weight, but increased visceral adipose tissue weight and adipocyte size. In addition, gene expression of leptin and IL6 in the visceral adipose tissue of zinc-supplemented mice were higher than those of control mice. Moreover, serum level of leptin of the zinc-supplemented mice was twice as high as that of the control mice. Besides, phosphorylation level of AKT T308 was attenuated in the perirenal adipose tissue of zinc-supplemented mice. In comparison, the expression of macrophage marker genes and lipogenic genes were not affected by chronic zinc supplementation, but the protein levels of FAS and SCD1 decreased or tended to decrease in the perirenal adipose tissue of zinc-supplemented mice, as compared to the control mice. Our findings suggest that chronic high dose zinc supplementation induces visceral adipose tissue hypertrophy and impairs AKT signaling in perirenal adipose tissue.

## 1. Introduction

The trace element zinc is essential for human health as it plays an important role in growth, immunity, reproduction, inflammation, metabolism and gastrointestinal function [1,2,3,4,5]. Notably, zinc deficiency induces metabolic diseases, such as non-alcoholic fatty liver disease (NAFLD), insulin resistance, adipose tissue inflammation and hyperglycemia [6,7,8]. Zinc displays an insulin-like character, which can stimulate cellular insulin signaling [9]. Short-term zinc supplementation reduces blood glucose level in individuals with obesity and type 2 diabetes [10,11,12], as well as reverses alcohol-induced steatosis [13]. Moreover, it is also reported that zinc stimulates the adipose differentiation of preadipocytes in vitro [14]. Besides, Zhang et al. reported that zinc supplementation induced intramuscular adipocytes content in weaned piglets [15].

Adipose tissue is a storage pool for excess energy, while triglyceride is the major source of energy in adipose tissue [16]. On the one hand, when energy intake is in excess, adipocytes uptake glucose and lipids from the blood stream to synthetize triglyceride, which is subsequently stored in adipocytes, in a process known as lipogenesis. On the other hand, when the body is in a negative energy balance state, triglyceride is mobilized by hormones to release free fatty acid (FFA) and glycerol into the blood stream, which is also known as lipolysis. FFA is subsequently used by the body as an energy source, while glycerol is used for gluconeogenesis [17]. Additionally, adipose tissue is a major endocrine organ, which secretes cytokines and adipokines, including adiponectin, leptin, monocyte chemotactic protein 1 (MCP1), interleukin 6 (IL6) and tumor necrosis factor alpha (TNFα) [18]. Furthermore, adipose tissue is a target organ of insulin, which stimulates the glucose uptake and lipogenesis while inhibiting the lipolysis in adipocytes. Notably, the insulin sensitivity of adipocytes can be regulated by cytokines, trace elements and drugs [19]. Increased lipogenesis or decreased lipolysis results in adipocyte hypertrophy, which subsequently induces the secretion of inflammatory cytokines and impairs insulin sensitivity of adipose tissue [20]. Afterwards, the reduced insulin sensitivity of adipose tissue breaks the balance of systemic glucose metabolism, which manifests as hyperglycemia, insulin resistance and glucose intolerance [21,22].

Lipogenesis is regulated by lipogenic enzymes, such as fatty acid synthase (FAS), stearoyl-CoA desaturase-1 (SCD1) and acetyl-CoA carboxylase 1 (ACC1), whose expression is up-regulated by peroxisome proliferator-activated receptor gamma (PPARγ), CCAAT-enhancer-binding protein alpha (C/BPα) and sterol-regulatory element binding protein (SREBP1). In comparison, hormone-sensitive lipase (HSL) is the rate-limiting enzyme for lipolysis [17,23,24]. In vitro study has revealed that a high zinc level in the medium stimulates the expression of *SREBP1*, *FAS*, *SCD1* and *ACC1* in hepatocytes [25]. Moreover, in vivo study also demonstrates that zinc supplementation for 40 days increases the expression of *PPARγ*, *SREBP1*, *FAS*, *SCD1* and *ACC1*, while decreasing the expression of *HSL* in the intramuscular fat tissue [15]. However, the effects of long-term chronic zinc supplementation on fat metabolism in visceral adipose tissue have not been well elucidated.

In the current study, mice were supplemented with zinc sulfate in the drinking water for over 20 weeks. The results indicated that, as compared to the control group, chronic zinc supplementation increased serum zinc concentration, impaired systemic glucose clearance, increased visceral adipose tissue weight and adipocyte size, as well as stimulated the expression and secretion of leptin in visceral adipose tissue. In addition, phosphorylation level of protein kinase B (AKT) and protein levels of FAS and SCD1 were decreased in the zinc-supplemented perirenal adipose tissue. Our study suggested that long-term chronic over-dosage zinc intake might increase the risk of visceral adipose tissue hypertrophy.

## 2. Materials and Methods 

### 2.1. Animal Study

Animal study protocol (MICE2015012, 7 October 2015) was reviewed and approved by the Animal Care and Use Committee of Sichuan Agricultural University. All animal procedures were performed according to the National Institutes of Health guide for the care and use of Laboratory animals. 3-week-old C57BL/6 male mice were obtained from Vital River Laboratory Animal Technology Co. Ltd. (Beijing, China). The mice were allowed one week of acclimation in a pathogen- free room at the temperature of 22 °C and the humidity of 60%. Subsequently, they were randomly divided into 2 groups according to similar average body weight. One group was given spring water, and the other was given 30 ppm zinc-supplemented spring water (Supplementary Table S1) (132.4 mg/L zinc sulfate heptahydrate (Z0251, Sigma, Shanghai, China)). Both groups were fed with normal chow diet according to AIN93, which contained 38.3 ppm zinc (Supplementary Table S1) (Dashuo, Chengdu, China). Mice were free to access water and food. Food intake, water consumption, and body weight were measured every two weeks. At age of 25 weeks, mice were made to fast overnight. Body weight and tail-vein blood glucose levels (Blood glucose strips (5D-2) were purchased from Beijingyicheng, Beijing, China) were measured at 8 a.m. in the next morning. Then, mice were euthanized using carbon dioxide, followed by cervical dislocation. Serum was collected for further analysis. Perirenal and epididymal adipose tissue were rapidly dissected and weighed. One piece of fat tissue was fixed in 10% formalin for H&E, while the remaining was frozen at −80 °C for further analysis. 

### 2.2. Insulin Tolerance Test and Glucose Tolerance Test

For insulin tolerance test (ITT), 20 weeks old mice were deprived of food in the morning. 6 h later, mice were injected intraperitoneally with 0.5 U/kg insulin (Novo Nordisk, Beijing, China). Tail-vein blood glucose levels were measured 0, 15, 30, 45, 60 and 90 min after insulin injection.

For glucose tolerance test (GTT), 23 weeks old mice were deprived of food for 14 h (overnight). The next morning, mice were intraperitoneally injected with 1.5 g/kg dextrose (G7021, Sigma, Shanghai, China). Tail-vein blood glucose levels were measured at 0, 15, 30, 45, 60 and 90 min after dextrose injection.

### 2.3. Zinc Content Analysis

Zinc contents in the water and feed and copper levels in the serum were measured using the method of flame atomic absorption spectrometry (ContrAA, Analytik Jena, Jena, Germany). Serum zinc concentration was measured with a zinc detection kit (E011, Nanjing Jiancheng Bioengineering Institute, Nanjing, China) according to the manufacturer’s instruction.

### 2.4. Serum Metabolites Profile Analysis

Serum triglyceride and FFA levels were measured on an automatic biochemical analyzer (7020, HITACHI, Tokyo, Japan) with their analysis kits respectively according to the manufacturer’s instructions. Triglyceride kit (CH0105151) was purchased from Muccura (Chengdu, China), and FFA kit (GS191Z) was obtained from Beijing Strong Biotechnologies (Beijing, China).

### 2.5. Serum Hormone Levels Measurement

Serum insulin levels were measured with a mouse insulin ultrasensitive ELISA kit (80-INSMSU, ALPCO, Salem, MA, USA) according to the manufacturer’s instruction.

Serum leptin levels were measured with a mouse leptin ELISA kit (ezml-82, Millipore, Billerica, MA, USA) according to the manufacturer’s instruction.

Serum IL6 and MCP1 levels were measured with the respected ELISA kits (431301 and 432701, BioLegend, San Diego, CA, USA) according to the manufacturer’s instruction.

### 2.6. Fat Tissue Histology Staining

For H&E staining, fresh fat tissues were fixed in 10% formalin for 48 h, and then dehydrated and embedded in paraffin. Embedded tissues were sliced into 4 μm sections (RM2016, Leica, Shanghai, China). Sections were then dehydrated, stained with hematoxylin for 5 min, washed with ddH_2_O, and stained with eosin for 2 min. The sections were then dehydrated and mounted with a neutral resin onto slides. Images were captured on a microscope (TS100, Nikon, Tokyo, Japan) with a CCD (DS-U3, Nikon, Tokyo, Japan) using imaging software (NIS-Elements F3.2, Nikon, Tokyo, Japan).

Cell area was measured with the software of ImageJ (National Institutes of Health, Bethesda, USA). Briefly, 16 images from 4 mice of each group were used for cell area measurement, and 10 cells which stood for the average cell size of each image were used to calculate the average cell size. The cell area of 160 cells in total for each group of each tissue was used for average cell area calculation.

### 2.7. Cell Culture

Cell culture and adipose differentiation were performed as previously reported [26]. Briefly, 3T3-L1 preadipocytes (CL-173, American Type Culture Collection, Manassas, VA, USA) were cultured in basic medium (DMEM medium (11995040, Gibco, Shanghai, China) supplemented with 10% fetal bovine serum (10099-141, Gibco, Shanghai, China), 100 U/mL penicillin and 100 µg/mL streptomycin (10378016, Gibco) at 37 °C and 5% CO_2_. Once 75% confluence was reached, cells were subcultured into 12-well plate at 90% confluence. 2 days after 100% confluence, cells were treated with 1 µg/mL insulin (I5500, Sigma, Shanghai, China), 1 µM dexamethasone (D1881, Sigma, Shanghai, China) and 0.5 mM isobutyl methyl xanthine (IBMX) (I7018, Sigma, Shanghai, China) for 3 days. Cells were then maintained in 1 µg/mL insulin-supplemented basic medium. Medium was freshly changed 3 times in the next days. On day 9, fully differentiated adipocytes were washed 3 times with serum-free DMEM, followed by incubating in serum-free DMEM medium for 14 h. Cells were then treated with 50 µM zinc or the vehicle for 6 h. 

### 2.8. RNA Extraction and Real-Time PCR

RNA extraction and real-time PCR were performed as previously reported [27]. Briefly, 100 mg fat tissue powder was homogenized in 1 mL Trizol Reagent (15596018, Invitrogen, Shanghai, China) and RNA was extracted in accordance with the manufacturer’s instruction. The quality of RNA was assessed by agarose gel and the concentration was measured with a spectrophotometer (NanoDrop 2000, Thermo Scientific, Shanghai, China). 1 μg RNA was reverse-transcribed into cDNA with a reverse-transcription PCR kit according to the manufacturer’s instructions (RR037A, Takara, Dalian, China). Real-time PCR was conducted on a quantitative-PCR machine (7900HT, ABI, Carlsbad, CA, USA) with Power SYBR Green RT-PCR reagents (4368702, Thermo Fisher Scientific, Shanghai, China). The following reagent amounts were used for each reaction: forward primer, 300 nM; reverse primer, 300 nM; cDNA sample, 20 ng. The conditions used for PCR were: 95 °C for 10 min for 1 cycle, and then 40 cycles of 95 °C for 15 s followed by 60 °C for 1 min. The real time PCR data was analyzed by the 2-delta delta CT method with *β-actin* as the reference. The sequences of the primers are listed below.

*β-actin* forward GGCTGTATTCCCCTCCATCG and reverse CCAGTTGGTAACAATGCCATGT; *FAS*, forward GGCTCTATGGATTACCCAAGC and reverse CCAGTGTTCGTTCCTCGGA; *SCD1*, forward CCTACGACAAGAACATTCAATCCC and reverse CAGGAACTCAGAAGCCCAAAGC; *CD11c*, forward CTGGATAGCCTTTCTTCTGCTG and reverse GCACACTGTGTCCGAACTCA; *F4/80*, forward TGACTCACCTTGTGGTCCTAA and reverse CTTCCCAGAATCCAGTCTTTCC; *MCP1*, forward TTAAAAACCTGGATCGGAACCAA and reverse GCATTAGCTTCAGATTTACGGGT; *Leptin*, forward GAGACCCCTGTGTCGGTTC and reverse CTGCGTGTGTGAAATGTCATTG; *IL6*, forward TAGTCCTTCCTACCCCAATTTCC and reverse TTGGTCCTTAGCCACTCCTTC; *Glut4*, forward ACCGGATTCCATCCCACAAG and reverse TCCCAACCATTGAGAAATGATGC; *PPARγ*, forward GGAAGACCACTCGCATTCCTT and reverse TCGCACTTTGGTATTCTTGGAG; *C/EBPα*, forward CAAGAACAGCAACGAGTACCG and reverse GTCACTGGTCAACTCCAGCAC; *SREBP1*, forward AACTGCCCATCCACCGACTC and reverse ATTGATAGAAGACCGGTAGCGC; *ACC1*, forward CGGACCTTTGAAGATTTTGTCAGG and reverse GCTTTATTCTGCTGGGTGAACTCTC; *PLIN*, forward CGTGGAGAGTAAGGATGTCAATG and reverse GGCTTCTTTGGTGCTGTTGTAG; *HSL*, forward TGAAGCCAAAGATGAAGTGAGAC and reverse CTTGACTATGGGTGACGTGTAGAG.

### 2.9. Western Blot Analysis

Western blot analysis was performed as previously reported [27]. For the preparation of protein lysates, 100 mg fat tissue powder was homogenized in 1 mL cell lysis buffer (P0013C, Beyotime Biotechnology, Shanghai, China) supplemented with protease inhibitor cocktail (04693132001, Roche, Mannheim, Germany) on a homogenizer. The concentration of protein in the supernatant was measured with a BCA Protein Assay Kit (23250, Thermo, Shanghai, China). 100 μg protein was used to prepare an electrophoresis sample with loading buffer (1610747, BioRad, Shanghai, China) in a volume of 30 μL for each sample. Proteins were separated on 12% polyacrylamide gel, and then transferred onto PVDF membranes (1620177, BioRad, Shanghai, China). The membranes were blocked in 1% BSA/1 × TBST for 1 h at room temperature, followed by incubation with the appropriate primary antibodies (1 µg/mL) overnight. pAKT T308 (4056), AKT (9272), PPARγ (2443), pHSL (3891), SCD1 (2794) and tubulin (3873) antibodies were obtained from Cell Signaling Technology (Shanghai, China); HSL (sc-74489) and FAS (sc-48357) antibody was obtained from Santa Cruz (Shanghai, China). After thorough washing, membranes were incubated with appropriate horseradish peroxidase-linked secondary antibodies (7074 and 7076, CST) (1:2000 dilution in 5% milk/1 × TBST) for 1 h. After further thorough washing, protein signals were detected by ECL western blotting detection reagent (1705060, BioRad, Shanghai, China) on a Molecular Imager ChemiDoc XRS+ System (BioRad). Blots were quantified with ImageJ software (National Institutes of Health).

### 2.10. Statistical Analysis

Data were analyzed using the SAS 9.3 software (SAS Institute Inc., Cary, NC, USA). The normality and homogeneity of variances of data were firstly tested with univariate test. Independent t-test was used to compare the difference between two groups with normal distribution data, while non-Gaussian and heterogeneous data were analyzed using non-parametric analysis. One-way repeated measures ANOVA was used to analyze the statistical difference of water consumption, food intake, GTT and ITT; one-way ANOVA was applied to analyze the effect of zinc sulfate on gene expression in 3T3-L1 adipocytes, when two-way ANOVA was used to analyze the effect of insulin and zinc on AKT phosphorylation in 3T3-L1 adipocytes. Post-hoc analysis was then applied. Results were presented as mean ± SEM. Statistical significance was determined at *P* < 0.05.

## 3. Results

### 3.1. Chronic Zinc Supplementation Induced Glucose Intolerance

**Figure 1:**
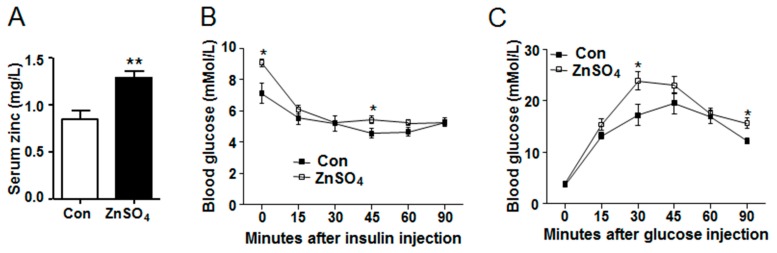
Chronic zinc supplementation impaired systemic insulin sensitivity and glucose clearance. (**A**) Serum zinc levels (*N* = 8 for each group) (*P* = 0.0017); (**B**) Insulin tolerance test (*P*_zinc_ = 0.1007; *P*_time_ < 0.0001; *P*_zincXtime_ = 0.0012; *P*_0_ = 0.0174; *P*_45_ = 0.0429) (*N* = 7 for each group); (**C**) Glucose tolerance test (*P*_zinc_ = 0.0318; *P*_time_ < 0.0001; *P*_zincXtime_ = 0.0621; *P*_30_ = 0.0278; *P*_90_ = 0.0131) (*N* = 7 for each group). Data were shown as mean ± SEM. Con, control group; ZnSO_4_, zinc sulfate-supplemented group. * *P* < 0.05, ** *P* < 0.01 ZnSO_4_ vs. Con.

To investigate the effect of long-term chronic zinc supplementation on adipose metabolism, mice were supplemented with zinc sulfate in the drinking water for 20 weeks. Results showed that zinc-supplemented mice had comparable water consumption and food intake to those of control mice during the experimental period, except that zinc-supplemented mice consumed more water at the age of 9 than the control mice (*P* = 0.0226) (Supplementary Figure S1A,B and Tables S2 and S3). As was expected, serum zinc concentration in zinc-supplemented mice was significantly higher than that in the control mice (*P* = 0.0017) (Figure 1A and Supplementary Table S4). At the same time, serum copper levels were similar between the zinc-supplemented group and control group, while the zinc/copper ratio was higher in the zinc-supplemented mice than that in the control mice (*P* = 0.0462) (Supplementary Figure S1C,D and Table S4). Blood glucose level was then measured, which revealed that zinc-supplemented mice had similar blood glucose levels to the control mice at both fed and fasting state (Supplementary Figure S2A,B and Table S5). Moreover, serum insulin remained unchanged in zinc-supplemented mice compared with that in the control mice (Supplementary Figure S2C and Supplementary Table S6). However, zinc-supplemented mice had higher blood glucose levels after 6 h fasting and 45 min of exogenous insulin challenge, as compared to the control mice (*P* = 0.0174 and *P* = 0.0429) (Figure 1B and Supplementary Table S7). In addition, zinc-supplemented mice had slower glucose clearance rate (*P* = 0.0318) and higher blood glucose levels 30 and 90 min after exogenous glucose administration (*P* = 0.0278 and *P* = 0.0131) (Figure 1C and Supplementary Table S8). These data indicated that chronic high dose zinc supplementation in the drinking water did not affect basal blood glucose or insulin levels, but impaired systemic glucose clearance rate after exogenous insulin and glucose challenge.

### 3.2. Chronic Zinc Supplementation Induced Visceral Adipose Tissue Hypertrophy

**Figure 2:**
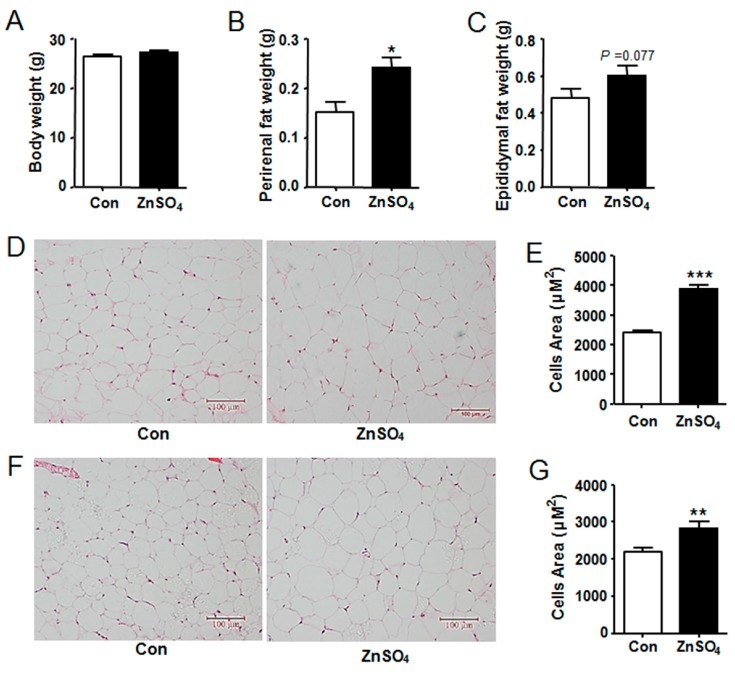
Chronic zinc supplementation induced visceral adipose tissue hypertrophy. (**A**) Body weight (*N* = 8 for each group); (**B**) The weight of perirenal adipose tissue (*N* = 8 for each group) (*P* = 0.0143); (**C**) The weight of epididymal adipose tissue (*N* = 8 for each group); (**D**) H&E staining images for perirenal fat pad; (**E**) Adipocyte size of perirenal adipose tissue (*N* = 160 cells from 4 mice for each group) (*P* < 0.0001); (**F**) H&E staining images for epididymal fat pad; (**G**) Adipocyte size of epididymal adipose tissue (*N* = 160 cells from 4 mice for each group) (*P* = 0.0012). Scale bars were equal to 100 μm. Data were shown as mean ± SEM. Con, control group; ZnSO_4_, zinc sulfate-supplemented group. * *P* < 0.05, ** *P* < 0.01, *** *P* < 0.001 ZnSO_4_ vs. Con.

Effects of zinc supplementation on body weight and tissue weight was subsequently examined. Results showed that zinc-supplemented mice had similar body weight to that of control mice during the experiment and at the time of harvest (Supplementary Figure S2D, Figure 2A and Supplementary Tables S9 and S10). Further study indicated that liver weight and subcutaneous adipose tissue weight were not altered by zinc supplementation, as compared to those of the control (Supplementary Figure S2E,F and Table S10). However, the weight of perirenal adipose tissue was increased by 60% in the zinc-supplemented mice, while that of epididymal adipose tissue tended to increase by 26%, relative to those in the control mice (*P* = 0.0143 and *P* = 0.0767) (Figure 2B,C and Supplementary Table S10). Similar results were obtained in a repeated experiment with another cohort of mice (data not shown). 

Both enlargement of adipocyte size and increase in adipocyte number result in adipose tissue hypertrophy. Thus, H&E staining was thereby performed to analyze the cell morphology, the results of which indicated that the adipocyte sizes of zinc-treated mice increased by 60% and 25% in perirenal fat tissue and epididymal fat tissue respectively, compared with those in the control mice (*P* < 0.0001 and *P* = 0.0012) (Figure 2D–G and Supplementary Table S11). These data demonstrated that chronic zinc supplementation induced visceral adipose tissue hypotrophy at least partially through enlarging adipocyte size in lean mice.

### 3.3. Chronic Zinc Supplementation Stimulated the Expression and Secretion of Leptin in Visceral Adipose Tissue

**Figure 3:**
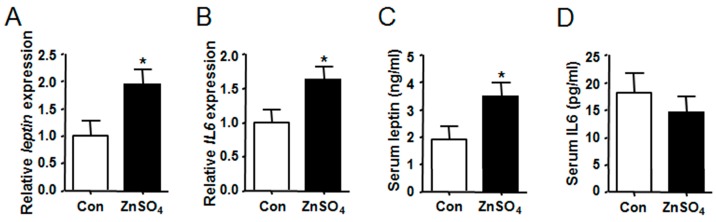
Chronic zinc supplementation stimulated the expression and secretion of leptin in perirenal adipose tissue. (**A**) The expression of *leptin* in perirenal adipose tissue (*P* = 0.0216); (**B**) The expression of IL6 in perirenal adipose tissue (*P* = 0.0297); (**C**) Serum leptin levels (*P* = 0.0420); (**D**) Serum IL6 levels. *N* = 8 for each group. Data were shown as mean ± SEM. Con, control group; ZnSO_4_, zinc sulfate-supplemented group. * *P* < 0.05 ZnSO_4_ vs. Con.

The expression of adipokine and cytokine genes in the adipose tissue were analyzed. Results indicated that both the expression of *leptin* and *IL6* were increased in the perirenal and epididymal adipose tissue of zinc-supplemented mice as compared to those in the control mice (*P* = 0.0329, *P* = 0.0297, *P* = 0.0328 and *P* = 0.0482) (Figure 3A,B and Supplementary Figure S3A,B and Tables S12 and S13). Moreover, serum levels of leptin and IL6 were subsequently analyzed, the results of which showed that serum leptin level of zinc-supplemented mice was twice as high as that of the control mice (*P* = 0.0420) (Figure 3C and Supplementary Table S6). However, serum level of IL6 remained unchanged (Figure 3D and Supplementary Table S6).

The effect of zinc on the expression of *leptin* and *IL6* in cultured adipocytes was also investigated. Results indicated that the expression of *leptin* and *IL6* were also increased by zinc sulfate or zinc chloride in cultured 3T3-L1 adipocytes (Supplementary Figure S4A–D and Tables S14 and S15).

### 3.4. Chronic Zinc Supplementation Attenuated AKT Phosphorylation in Perirenal Adipose Tissue

**Figure 4:**
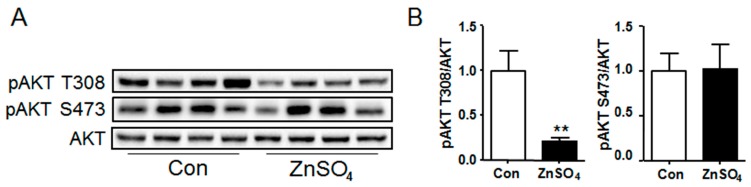
Chronic zinc supplementation attenuated the phosphorylation of AKT in perirenal adipose tissue. (**A**) Western blot bands of phosphorylation AKT and total AKT. Data represented 8 mice of 16 in total; (**B**) The quantification of phosphorylation AKT levels in perirenal adipose tissue (*N* = 8 for each group) (*P* = 0.0040 for pAKT T308). Data were shown as mean ± SEM. Con, control group; ZnSO_4_, zinc sulfate-supplemented group. ** *P* < 0.05 ZnSO_4_ vs. Con.

AKT signaling in the adipose tissue was subsequently detected. Results illustrated that the phosphorylation level of AKT T308 was outstandingly attenuated in the perirenal adipose tissue of zinc-supplemented mice compared with that of control mice (*P* = 0.0040), while that of AKT S473 remained unchanged (Figure 4A,B and Supplementary Table S16). However, phosphorylation level of either AKT T308 or S473 in the epididymal adipose tissue of zinc-supplemented mice was similar to that of control mice (Supplementary Figure S5A and Table S17). Meanwhile, the effect of zinc supplementation on AKT phosphorylation was also investigated in cultured adipocytes, the results of which revealed that zinc supplementation induced the phosphorylation of both AKT T308 and S473 in cultured adipocytes, both at basal and insulin-stimulating state (Supplementary Figure S5B and Table S18).

### 3.5. Chronic Zinc Supplementation Did Not Alter Macrophage Content in the Adipose Tissue

**Figure 5:**
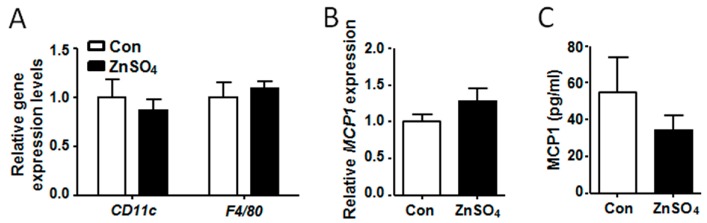
Chronic zinc supplementation did not alter the expression of macrophage marker genes and *MCP1* in perirenal adipose tissue. (**A**) The expression of *CD11c* and *F4/80* in perirenal adipose tissue; (**B**) The expression of *MCP1* in perirenal adipose tissue; (**C**) Serum MCP1 levels. Data were shown as mean ± SEM. Con, control group; ZnSO_4_, zinc sulfate-supplemented group.

It was reported that chronic macrophage infiltration induced inflammation and hypertrophy of adipose tissue [28]. Thus, the expression of macrophage marker genes was analyzed, the results of which showed that the expression of *CD11c* and *F4/80* in the visceral adipose tissues of chronic zinc-supplemented mice remained unchanged, as compared to the control mice (Figure 5A, Supplementary Figure S3C and Tables S12 and S13). MCP1 was reported to stimulated adipose tissue to recruit macrophages [28]. However, the expression of *MCP1* in the visceral adipose tissue of zinc-supplemented mice was similar to that of the control mice (Figure 5B and Supplementary Figure S3D and Tables S12 and S13). Further analysis indicated that serum MCP1 level in the zinc-supplemented mice was also comparable to that in the control mice (Figure 5C and Supplementary Table S6). These data demonstrated that chronic zinc supplementation did not alter microphage content in the visceral adipose tissue.

### 3.6. The Protein Levels of FAS and SCD1 Were Decreased in the Perirenal Adipose Tissue of Chronic Zinc-Supplemented Mice

**Figure 6:**
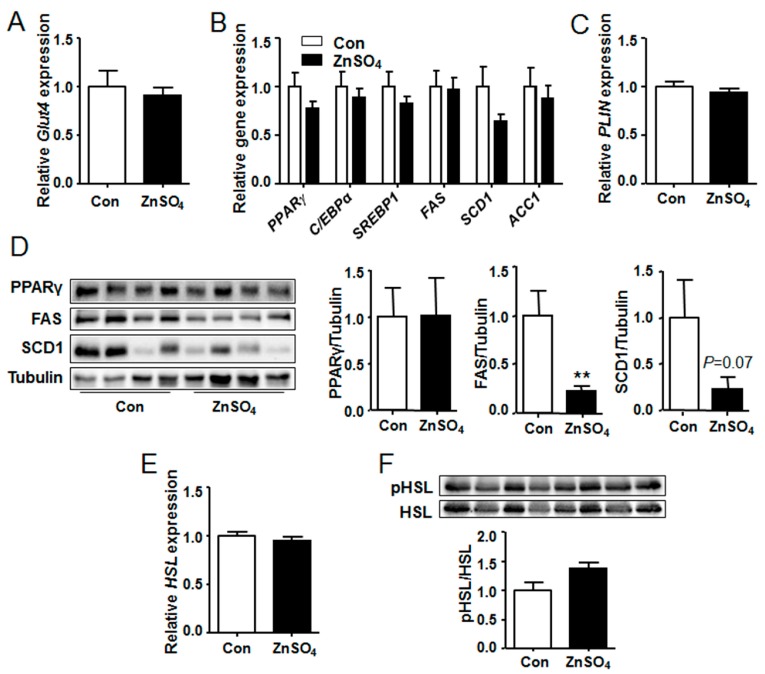
Chronic zinc supplementation did not alter the expression of lipogenic genes but decreased protein levels of FAS and SCD1 in perirenal adipose tissue. (**A**) The expression of *Glut4*; (**B**) The expression of lipogenic genes and their regulatory genes; (**C**) The expression of *PLIN*; (**D**) Protein levels of PPARγ, FAS and SCD1 (*P* = 0.0006 for FAS); (**E**) The expression of *HSL*; (**F**) Phosphorylation levels of HSL. *N* = 8 for each group. Data were shown as mean ± SEM. Western blots represented 8 mice of 16 in total. Con, control group; ZnSO_4_, zinc sulfate-supplemented group. ** *P* < 0.01 ZnSO_4_ vs. Con.

As the adipose tissue weight and the adipocyte size were increased in the zinc-supplemented mice, the expression of fat metabolic genes was then investigated. Results suggested that the expression of glucose transporter 4 (*Glut4*), the main regulator for adipocyte glucose uptake, remained unchanged in the perirenal fat and epididymal fat of zinc-supplemented mice, as compared with those of control mice (Figure 6A, Supplementary Figure S6A and Tables S12 and S13). Besides, the expression of lipogenic genes and lipid droplets coating gene were unchanged by zinc supplementation, as compared with those of the control group (Figure 6B,C and Supplementary Figure S6B,C, Tables S12 and S13). Furthermore, protein level of PPAR γ in the perirenal fat tissue remained unchanged by zinc supplementation (Figure 6D and Supplementary Table S16). However, compared with control mice, the protein level of FAS (*P* = 0.0006) was significantly decreased and that of SCD1 (*P* = 0.0723) tended to decrease in the perirenal adipose tissue of zinc supplemented-mice (Figure 6D and Supplementary Table S16). Moreover, protein levels of FAS and SCD1 in epididymal adipose tissue of zinc-supplemented mice were similar to those of the control mice (data not shown).

Both lipolysis and lipogenesis regulate adipose deposition in the adipose tissue [17]. Consequently, gene expression and protein phosphorylation levels of lipolysis regulator HSL were analyzed. Results showed that both gene expression and phosphorylation level of HSL in the visceral adipose tissue of zinc-supplemented mice were similar to those of the control mice (Figure 6E,F and Supplementary Figure S6D,E and Tables S12, S13, S16 and S17). These data indicated that lipolysis might be not affected by chronic zinc supplementation.

### 3.7. Chronic Zinc Supplementation Did Not Alter the Metabolic Profiles of Serum Lipids

**Figure 7:**
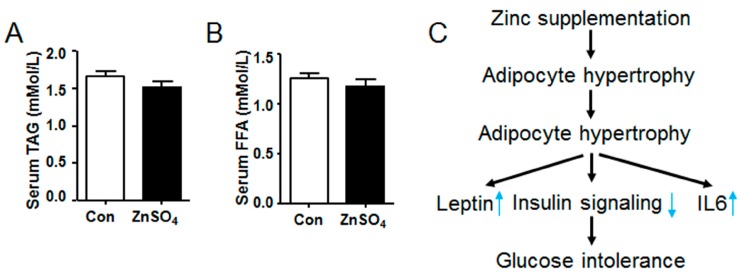
Chronic zinc supplementation did not affect serum levels of triglyceride and free fatty acid. (**A**) Serum triglyceride levels; (**B**) Serum free fatty acid levels; (**C**) The illustration of chronic zinc supplementation inducing adipose tissue hypertrophy. *N* = 8 for each group. Data were shown as mean ± SEM. Con, control group; ZnSO_4_, zinc sulfate-supplemented group.

Metabolic profiles of serum lipids were further investigated, which indicated that both serum TAG and serum FFA levels remained unchanged in the zinc-supplemented mice relative to those in the control mice (Figure 7A,B and Supplementary Table S19). 

## 4. Discussion

It is reported that zinc stimulates insulin sensitivity and induces adipogenesis in adipocytes. However, it is shown in the current study that long-term chronic zinc supplementation in the drinking water impairs systemic glucose clearance rate, induces visceral adipocyte tissue hypertrophy and attenuates AKT signaling in perirenal adipose tissue. Meanwhile, zinc supplementation stimulates the expression of *leptin* and *IL6* in visceral adipose tissue while increasing serum leptin level (Figure 7C). Moreover, it is also found that protein levels of FAS and SCD1 are down-regulated in perirenal adipose tissue of chronic zinc-supplemented mice relative to those of control mice, though their gene expression are similar to those of control mice.

### 4.1. Correlation of Chronic Zinc Supplementation with Systemic Glucose Clearance

It has been reported that diet induced zinc deficiency impairs systemic insulin sensitivity, whereas acute zinc supplementation enhances insulin signaling and glucose deposition [29,30,31]. However, the study of Kim et al. has reported that zinc supplementation for eight weeks does not improve systemic insulin resistance in humans [32]. Besides, zinc supplementation for four weeks shows no effect on insulin sensitivity in healthy black and white early-adolescent girls [33]. Furthermore, ITT and GTT data of our study suggest that chronic zinc supplementation attenuates systemic insulin sensitivity and glucose clearance (Figure 1B,C). Further study on AKT phosphorylation levels indicates that chronic zinc supplementation attenuates AKT signaling in the perirenal adipose tissue (Figure 4), which may be the reason why chronic zinc supplementation impairs the systemic glucose clearance after exogenous insulin or glucose challenges as compared with the control group. Zinc stimulates insulin signaling in adipocytes (Supplementary Figure S5B), while acute zinc supplementation enhances insulin sensitivity in adipose tissue [31]. Thus, the impaired AKT signaling may be attributed to the secondary effect of adipose tissue hypertrophy (Figure 2). Difference in the effects of zinc supplementation on systemic insulin sensitivity and glucose clearance among different studies may be resulted from the different duration of zinc supplementation.

Increased blood glucose level stimulates the beta islet cells to secret insulin, which subsequently gives rise to insulin signaling in perirenal tissues, such as liver, adipose tissue and muscle. The induced insulin signaling stimulates glycogen synthesis in liver as well as stimulates glucose uptake in adipose tissue and muscle, thus reducing the blood glucose level to a normal level. On the other hand, the secretion of insulin in the beta islet cells is inhibited in the presence of low blood glucose level, whereas the secretion of glucagon is stimulated to induce glycogenolysis and gluconeogenesis. However, the glucose decreasing effect of insulin is diminished when the insulin sensitivity of perirenal tissue is impaired, which results in insulin resistance and hyperinsulinemia [34]. The results of insulin tolerance and glucose intolerance should be at least partially ascribed to the impaired insulin sensitivity of adipose tissue (Figure 1B,C), since the serum insulin levels are similar between the zinc-supplemented mice and the control mice (Figure S2C).

Blood glucose is mainly obtained from glycogenolysis in the first 12 h of fasting, while gluconeogenesis is the major source of blood glucose after 12 h of fasting [35]. Thus, the finding that zinc decreased the blood glucose levels after 6 h of fasting, but not at 14 h of fasting suggests that chronic zinc supplementation may impair glycogenolysis in the liver. However, the exact mechanism needs to be explored in future study.

### 4.2. Correlation of Chronic Zinc Supplementation with Adipose Accumulation in Visceral Adipose Tissue

Zinc is a stimulator for insulin signaling, which mimics several actions of insulin [31]. Ghosh et al. reported that zinc-chelated vitamin C stimulated the adipogenesis in preadipocytes [14]. In addition, it is demonstrated in the current study that long-term chronic zinc supplementation induces adipose accumulation of the visceral adipose tissue in mice (Figure 2). Zhang et al. reported that dietary zinc supplementation increased intramuscular adipose deposition in piglets, which supports our results [15]. Our data reveals that the expression of glucose transporter gene and lipogenic genes are not affected by chronic zinc supplementation (Figure 6A,B and Supplementary Figure S6A,B). Therefore, the increased adipose accumulation may be independent of lipogenesis at the time of harvest. At the same time, the expression and phosphorylation level of HSL remain unchanged in the visceral adipose tissue of zinc-supplemented mice (Figure 6E,F and Supplementary Figure S6D,E). Consequently, the increased adipose deposition is probably independent of lipolysis. Besides, serum TAG and FFA levels are similar in both control and zinc-supplemented mice (Figure 7), further supporting the observation that lipolysis in the visceral adipose tissue is not altered by chronic zinc supplementation. Thus, the increased adipogenesis at the early stage of zinc supplementation may lead to the hypertrophy of visceral adipose tissue at the late stage of zinc supplementation. Nevertheless, further study is needed to elucidate the exact mechanism by which mice accumulate more visceral adipose tissue than control mice after long-term chronic zinc supplementation.

Compared with control mice, the gene expression of *FAS* and *SCD1* remains unchanged in the chronic zinc-supplemented mice. Therefore, the decreased protein levels of FAS and SCD1 (Figure 6D) may be regulated by chronic high dose zinc supplementation in a post-translational manner. However, the precise mechanism by which chronic high dose zinc supplementation down-regulates the protein levels of FAS and SCD1 in adipose tissue remains to be elucidated in further studies.

### 4.3. Correlation of Chronic Zinc Supplementation with Adipokines and Cytokines

Leptin is one of the most abundant adipokines secreted by adipocytes [36], which is positively corelated with obesity, diabetes and insulin resistance [37]. The expression of leptin in the visceral adipose tissue, together with the serum leptin level, is remarkably increased in the chronic zinc-supplemented mice (Figure 3A,C). In addition, it is indicated in our in vitro study that zinc also stimulates the expression of leptin in 3T3-L1 adipocytes (Supplementary Figure S4A,B). These findings are supported by the report of Baltaci et al., which demonstrates that zinc deficiency for six weeks leads to decreased plasma leptin level, whereas zinc supplementation for six weeks significantly increases plasma leptin level [38]. In addition, Ott and Shay also reported that zinc deficiency reduced the expression and secretion of leptin in cultured rat adipocytes [39], which was similar to the findings of our study. However, it is also reported in some studies that short-term zinc supplementation reduces serum leptin level, whereas zinc deficiency for three weeks increases circulatory leptin level [7,40]. The contradictory results from different studies may be related to the duration of zinc deficiency or zinc supplementation. However, the exact mechanism by which zinc upregulates the expression of leptin should be elucidated in further studies.

Inflammation impairs the insulin sensitivity of adipose tissue [28,41]. The expression of IL6 is increased in the visceral adipose tissue of chronic zinc-supplemented mice (Figure 3B and Supplementary Figure S3B), which may also give rise to insulin intolerance in zinc-supplemented mice. Furthermore, macrophage infiltration into the adipose tissue also accounts for one of the causes of adipose inflammation and insulin resistance [28,41]. However, our findings demonstrate that chronic zinc supplementation does not alter macrophage content in the adipose tissue (Figure 5). In addition, leptin is reported to be a pro-inflammatory factor, which stimulates the expression of inflammatory factors [42]. This suggests that chronic zinc supplementation may stimulate the expression of inflammation factors (such as IL6) in adipocytes and macrophages through leptin.

Taken together, it is indicated in our study that chronic high dose zinc supplementation will increase the risk of visceral adipose tissue hypertrophy and systemic glucose intolerance. Thus, chronic high dose zinc supplementation may be harmful for health. Therefore, zinc supplementation should be carried out in controlled time and dosage. Moreover, further studies will be focused on the mechanism by which chronic zinc supplementation induces visceral adipose tissue hypertrophy as well as the different effects of short-term and long-term zinc supplementation on fat metabolism.

## Supplementary Materials

The following are available online at www.mdpi.com/2072-6643/9/10/1138/s1, Figure S1: Water consumption and food intake of mice during the experiment; Figure S2: Blood glucose levels, body weight and tissue weight; Figure S3: Gene expression in epididymal adipose tissue; Figure S4: Gene expression in 3T3-L1 adipocytes; Figure S5: Phosphorylation levels of AKT in epididymal adipose tissue and 3T3-L1 adipocytes; Figure S6: The expression of fat metabolic genes in epididymal adipose tissue; Table S1: The concentration of zinc in the drinking water; and Tables S2–S19: Data for all the figures.
