# Supplementary material for: Chronic High Dose Zinc Supplementation Induces Visceral Adipose Tissue Hypertrophy without Altering Body Weight in Mice
Source: Nutrients. 2017 Oct 18;9(10):1138. doi: 10.3390/nu9101138 (PMC5691754; doi:10.3390/nu9101138)
Supplement: Supplementary file 1 [file nutrients-09-01138-s001.pdf]

# Chronic high dose zinc supplementation induces visceral adipose tissue hypertrophy without altering body weight in mice

## Supplementary Materials

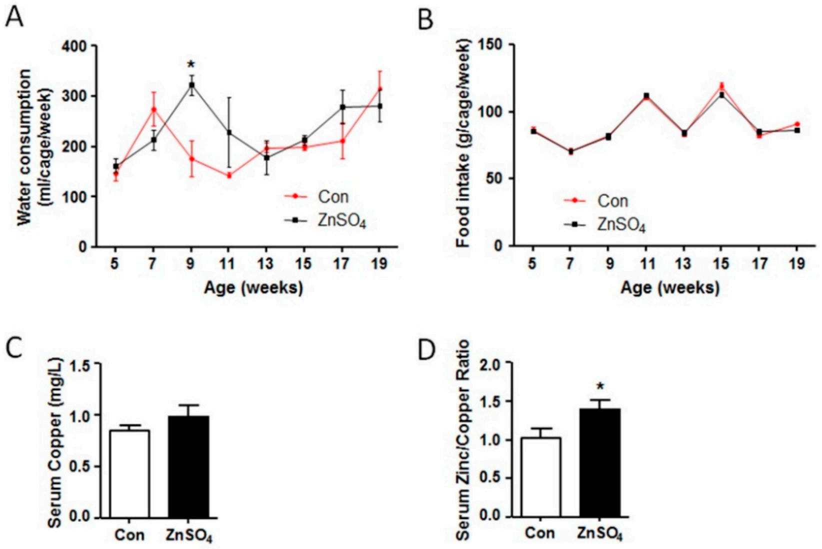


**Figure S1.** Water consumption and food intake of mice during the experiment. 12 mice were housed in 3 cages at 4 mice per cage. At the beginning of the week, the initial food weight and water volume for every cage were recorded. And then, the left food weight and water volume were measured at the end of the week. Food intake and water consumption were calculated as: initial food weight or water volume-left food weight or water volume. (**A**) Water consumption ($P_{zinc}$=0.1960; $P_{time}$=0.0004; $P_{zinc \times time}$=0.0290) (N=3 for each group); (**B**) Food intake ($P_{zinc}$=0.5815; $P_{time}$<0.0001; $P_{zinc \times time}$=0.0918) (N=3 for each group). (**C**) Serum copper levels at harvest (N=3 for each group). (**D**) Serum zinc/copper ratio at harvest (*P*=0.0462) (N=3 for each group). Data were shown as mean ± SEM. Con, control group; $ZnSO_4$, zinc sulfate-supplemented group. * *P*<0.05 $ZnSO_4$ vs. Con.

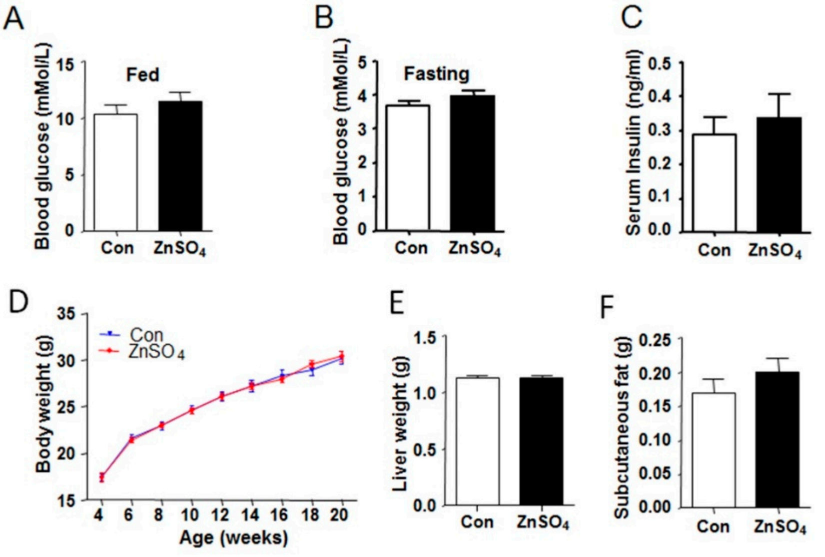


**Figure S2.** Blood glucose levels, body weight and tissue weight (N=8 for each group). (**A**) Blood glucose levels at fed state (N=8 for each group); (**B**) Blood glucose levels at fasting state (N=8 for each group); (**C**) Serum insulin levels (N=8 for each group); (**D**) Body weight during the experiment (N=12 for each group); (**E**) Liver weight

(N=8 for each group); (F) The weight of subcutaneous adipose tissue (N=8 for each group). Data were shown as mean ± SEM. Con, control group; $ZnSO_4$, zinc sulfate-supplemented group.

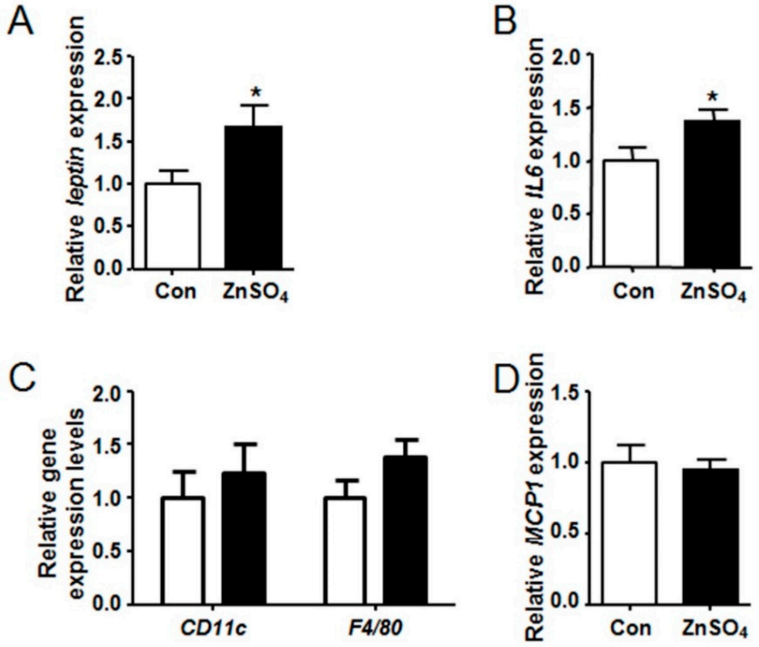


**Figure S3.** Gene expression in epididymal adipose tissue. (**A**) The expression of *leptin* ($P$=0.0328); (**B**) The expression of *IL6* ($P$=0.0482); (**C**) The expression of macrophage marker genes; (**D**) The expression of *MCP1*. N=8 for each group. Data were shown as mean ± SEM. Con, control group; $ZnSO_4$, zinc sulfate-supplemented group. * $P<0.05$ ZnSO4 vs. Con.

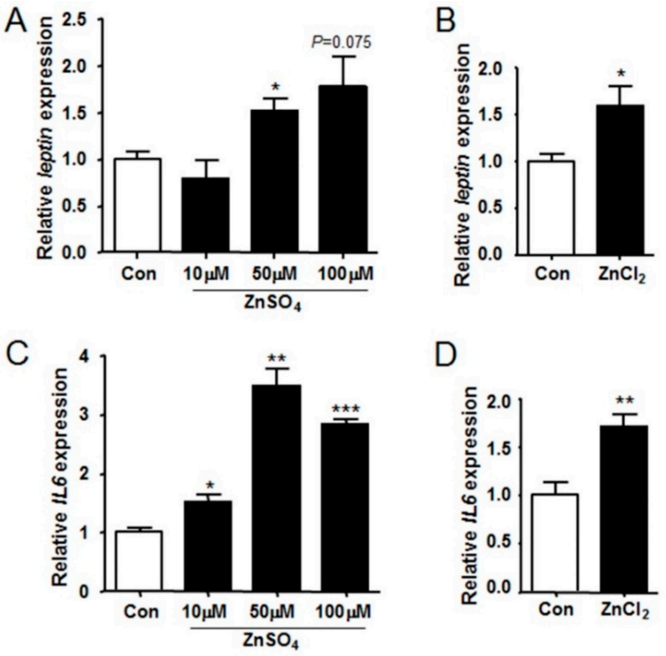


**Figure S4.** Gene expression in 3T3-L1 adipocytes. 3T3-L1 adipocytes were incubated in serum-free DMEM medium for 14 h. Cells were then treated with 50 μM zinc or the vehicle for 6 h. (**A**) The expression of *leptin* after zinc sulfate treatment ($P_{zinc}$=0.0271, $P_{50\mu M}$=0.0238); (**B**) The expression of *leptin* after zinc chloride treatment ($P$=0.0001); (**C**) The expression of *IL6* after zinc sulfate treatment ($P_{zinc}$<0.0001, $P_{10\mu M}$=0.0250, $P_{50\mu M}$=0.0013, $P_{100\mu M}$=0.0001); (**D**) The expression of *IL6* after zinc chloride treatment ($P$=0.0001). N=3 for each treatment. Data

were shown as mean ± SEM. Results represented one of three repeated experiments. Con, control group; $ZnSO_4$, zinc sulfate-treated group; $ZnCl_2$, zinc chloride-treated group. * *P*<0.05, ** *P*<0.01, *** *P*<0.001 Zinc vs. Con.

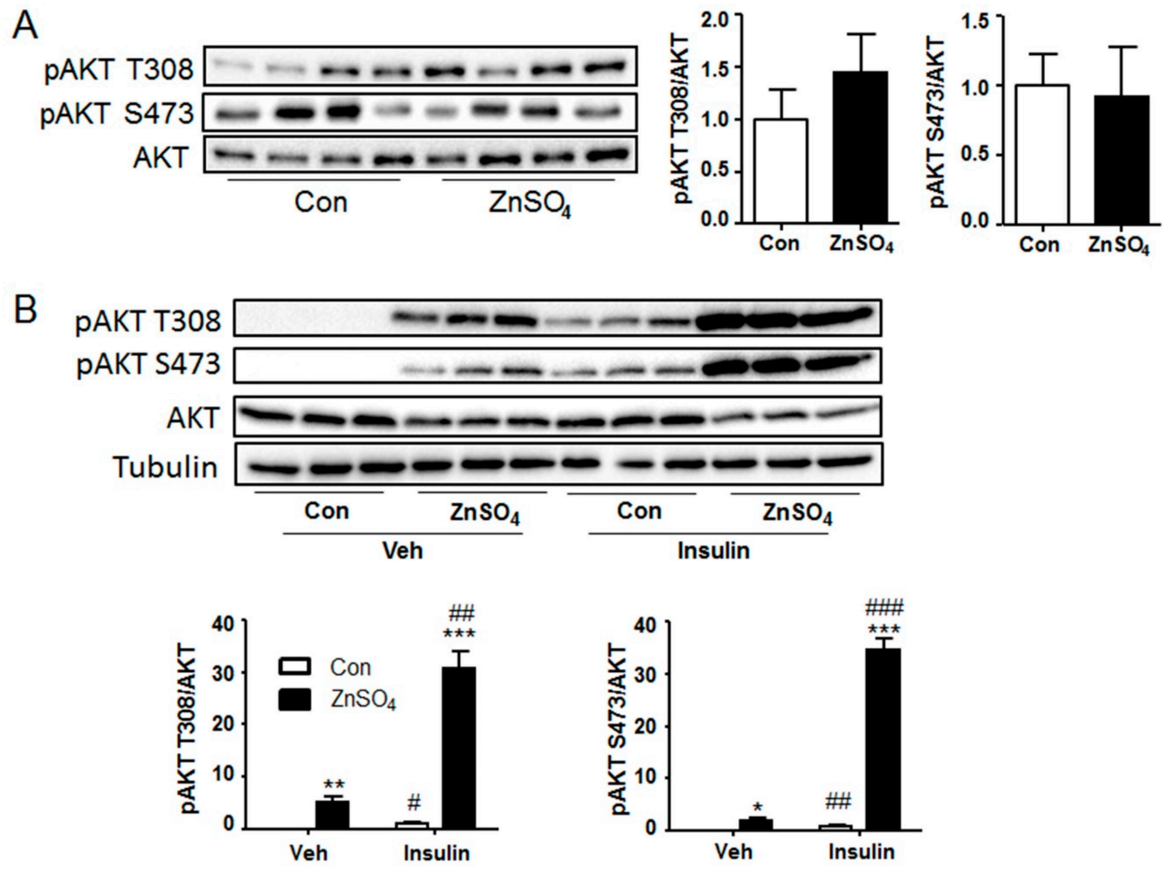


**Figure S5.** Phosphorylation level of AKT in epididymal adipose tissue and 3T3-L1 adipocytes. (**A**) Phosphorylation levels of AKT T308 and S473 in epididymal adipose tissue (N=8 for each group). Western blot bands represented 8 mice of 16 in total. (**B**) Phosphorylation levels of AKT T308 and S473 in 3T3-L1 adipocytes (N=3 for each group) ($P_{zinc}$<0.0001, $P_{Insulin}$<0.0001, $P_{Interaction}$=0.0001 for pAKT T308 and $P_{zinc}$<0.0001, $P_{Insulin}$<0.0001, $P_{Interaction}$<0.0001 for pAKT S473). 3T3-L1 adipocytes were incubated in serum-free DMEM medium for 14 h, followed by 6 h treatment with zinc sulfate or control. Cells were then treated with zinc sulfate or control plus 100 nM insulin for 5 min. Results represented one of three repeated experiments. Data were shown as mean ± SEM. Con, control mice or cells; $ZnSO_4$, zinc sulfate-treated mice or cells; Veh, the vehicle for insulin. * *P*<0.05, ** *P*<0.01, *** *P*<0.001 $ZnSO_4$ vs. Con; # *P*<0.05, ## *P*<0.01, ### *P*<0.001 $ZnSO_4$.

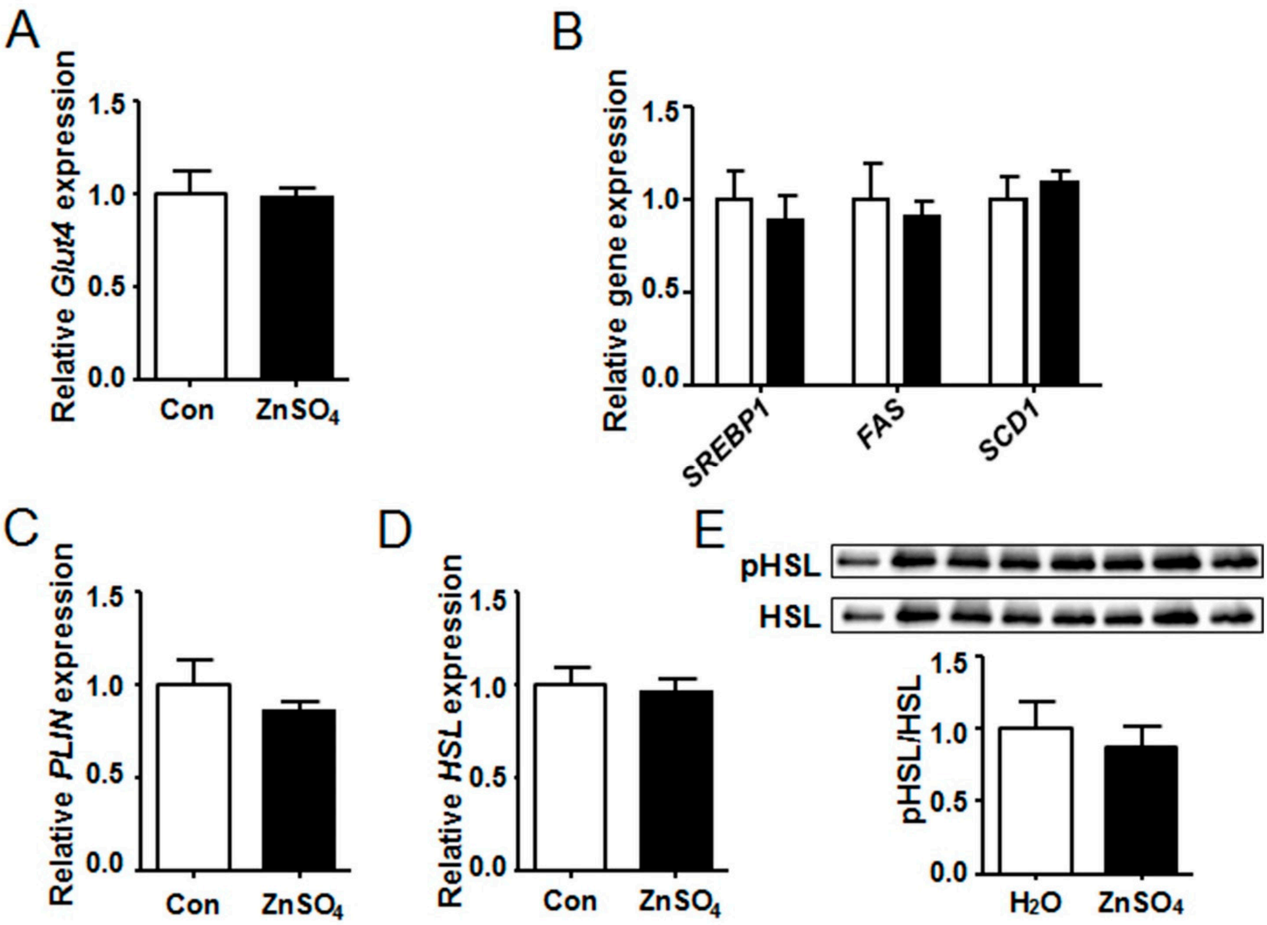


**Figure S6.** The expression of lipids metabolic genes in epididymal adipose tissue. (**A**) The expression of *Glut4*; (**B**) The expression of lipogenic genes; (**C**) The expression of *PLIN*; (**D**) The expression of *HSL*. (**E**) The phosphorylation levels of HSL in epddidymal adipose tissue. N=8 for each group. Data were shown as mean ± SEM. Con, control group; $ZnSO_4$, zinc sulfate-supplemented group.

**Table S1** Concentration of zinc in the drinking water

| Samples | Concentration of Zinc[@] | *P* value |
|---|---|---|
| Spring water | 0.002±0.0001 (mg/L) | <0.0001 |
| $ZnSO_4$ supplemented spring water | 29.651±0.892 (mg/L) | |
| Feed | 38.467±2.437 (mg/kg) | — |

@, samples from random three times were processed to analyze the concentration of zinc.

**Table S2** Water consumption (ml/cage/week)

| Age (weeks) | Control (mean±SEM) | Zinc (mean±SEM) | *P* value | | | |
|---|---|---|---|---|---|---|
| | | | post-hoc | Zinc | Age | Zinc X Age |
| 5 | 147±16 | 162±14 | 0.5344 | 0.1960 | 0.0004 | 0.0290 |
| 7 | 274±33 | 213±20 | 0.1862 | | | |
| 9 | 177±35 | 322±20* | 0.0226 | | | |
| 11 | 143±6 | 228±70 | 0.2920 | | | |
| 13 | 198±9 | 179±34 | 0.6153 | | | |
| 15 | 200±8 | 213±9 | 0.3235 | | | |
| 17 | 212±36 | 278±33 | 0.2468 | | | |
| 19 | 315±36 | 280±32 | 0.5039 | | | |

N=3 for each group, * *P*<0.05 Zinc vs Control.

**Table S3** Food intake (g/cage/week)

| Age (weeks) | Control (mean±SEM) | Zinc (mean±SEM) | *P* value | | | |
|---|---|---|---|---|---|---|
| | | | post-hoc | Zinc | Age | Zinc X Age |
| 5 | 86.50±2.58 | 85.70±0.81 | 0.7802 | 0.5815 | <0.0001 | 0.0918 |
| 7 | 70.82±2.10 | 70.71±0.42 | 0.9615 | | | |
| 9 | 82.49±1.46 | 81.57±2.39 | 0.7592 | | | |
| 11 | 111.14±1.98 | 112.52±0.45 | 0.5340 | | | |
| 13 | 84.36±2.17 | 84.83±1.90 | 0.8785 | | | |
| 15 | 119.38±2.72 | 113.04±2.18 | 0.1431 | | | |
| 17 | 82.41±1.66 | 85.44±1.97 | 0.3058 | | | |
| 19 | 91.48±0.94 | 86.74±1.72 | 0.0724 | | | |

N=3 for each group.

**Table S4** Serum zinc and copper levels

| | Control (mean±SEM) | Zinc (mean±SEM) | *P* value |
|---|---|---|---|
| Serum Zinc levels (mg/L) | 0.85±0.09 | 1.29±0.07* | 0.0017 |
| Serum Copper levels (mg/L) | 0.85±0.05 | 0.99±0.11 | 0.2927 |
| Serum Zinc/Copper ratio | 1.02±0.12 | 1.39±0.12* | 0.0462 |

N=8 for each group, * *P*<0.05 Zinc vs Control.

**Table S5** Blood glucose levels

| | Control (mean±SEM) | Zinc (mean±SEM) | *P* value |
|---|---|---|---|
| Blood glucose at fed (mMol/L) | 10.34±0.80 | 11.40±0.86 | 0.3775 |
| Blood glucose at fasting (mMol/L) | 3.77±0.13 | 3.93±0.14 | 0.4223 |

N=8 for each group.

**Table S6** Serum cytokine levels

| | Control (mean±SEM) | Zinc (mean±SEM) | *P* value |
|---|---|---|---|
| Leptin (ng/ml) | 1.91±0.48 | 3.48±0.51* | 0.0420 |
| IL6 (pg/ml) | 18.17±3.56 | 14.56±2.81 | 0.3812 |
| MCP1 (pg/ml) | 54.87±19.17 | 34.20±8.04 | 0.4493 |
| Insulin (ng/ml) | 0.29±0.05 | 0.33±0.07 | 0.6040 |

N=8 for each group, * *P*<0.05 Zinc vs Control.

**Table S7** Blood glucose levels during ITT (mMol/L)

| | | | *P*-value | | | |
|---|---|---|---|---|---|---|
| Minutes | Control (mean±SEM) | Zinc (mean±SEM) | post-hoc | Zinc | Time | Zinc X Time |
| 0 | 7.11±0.66 | 9.06±0.25* | 0.0174 | 0.1007 | <0.0001 | 0.0012 |
| 15 | 5.53±0.38 | 6.07±0.30 | 0.2847 | | | |
| 30 | 5.20±0.49 | 5.09±0.20 | 0.8320 | | | |
| 45 | 4.49±0.26 | 5.36±0.28* | 0.0429 | | | |
| 60 | 4.66±0.28 | 5.24±0.17 | 0.1013 | | | |
| 90 | 5.24±0.19 | 5.27±0.25 | 0.9293 | | | |

N=7 for each group, * *P*<0.05 Zinc vs Control.

**Table S8** Blood glucose levels during GTT (mMol/L)

| | | | *P*-value | | | |
|---|---|---|---|---|---|---|
| Minutes | Control (mean±SEM) | Zinc (mean±SEM) | post-hoc | Zinc | Time | Zinc X Time |
| 0 | 3.67±0.14 | 3.94±0.15 | 0.2133 | 0.0318 | <0.0001 | 0.0621 |
| 15 | 13.20±0.62 | 15.37±1.06 | 0.1025 | | | |
| 30 | 17.21±1.99 | 23.87±1.76* | 0.0278 | | | |
| 45 | 19.56±2.08 | 22.99±1.65 | 0.2214 | | | |
| 60 | 16.89±1.36 | 17.50±1.01 | 0.7224 | | | |
| 90 | 12.19±0.53 | 15.63±1.06* | 0.0131 | | | |

N=7 for each group, * *P*<0.05 Zinc vs Control.

**Table S9** Body weight during experiment (g)

| Age (weeks) | Control (mean±SEM) | Zinc (mean±SEM) | *P* valuve | | | |
|---|---|---|---|---|---|---|
| | | | post-hoc | Zinc | Age | Zinc X Age |
| 4 | 17.36±0.40 | 17.37±0.51 | 0.9857 | | | |
| 6 | 21.53±0.39 | 21.37±0.33 | 0.7641 | | | |
| 8 | 22.89±0.42 | 22.94±0.26 | 0.9233 | | | |
| 10 | 24.61±0.40 | 24.52±0.28 | 0.8639 | | | |
| 12 | 26.07±0.48 | 26.09±0.36 | 0.9761 | 0.9665 | <0.0001 | 0.9023 |
| 14 | 27.16±0.59 | 27.14±0.34 | 0.9767 | | | |
| 16 | 28.22±0.71 | 27.84±0.29 | 0.6219 | | | |
| 18 | 28.92±0.66 | 29.51±0.36 | 0.4406 | | | |
| 20 | 30.16±0.66 | 30.34±0.54 | 0.8383 | | | |

N=12 for each group.

**Table S10** Body weight and tissue weight at harvesting

| | Control (mean±SEM) | Zinc (mean±SEM) | *P* valuve |
|---|---|---|---|
| Body weight (g) | 26.34±0.43 | 27.17±0.40 | 0.1814 |
| Liver weight (g) | 1.13±0.02 | 1.13±0.02 | 0.9327 |
| Perirenal fat weight (g) | 0.15±0.02 | 0.24±0.02* | 0.0143 |
| Epididymal fat weight (g) | 0.48±0.05 | 0.60±0.05 | 0.0767 |
| Subcutaneous fat weight (g) | 0.17±0.02 | 0.20±0.02 | 0.3032 |

N=8 for each group, * $P<0.05$ Zinc vs Control

**Table S11** Adipocyte size (μm²)

| | Control (mean±SEM) | Zinc (mean±SEM) | *P* value |
|---|---|---|---|
| Perirenal adipocytes | 2410±83 | 3898±102* | <0.0001 |
| Epididymal adipocytes | 2184±122 | 2812±149* | 0.0012 |

N=160 for each group, * $P<0.05$ Zinc vs Control.

**Table S12** Relative gene expression in the perirenal adipose tissue

| Gene | Control (mean±SEM) | Zinc (mean±SEM) | *P* value |
|---|---|---|---|
| *Leptin* | 1.00±0.26 | 1.95±0.26* | 0.0216 |
| *IL6* | 1.00±0.18 | 1.62±0.19* | 0.0297 |
| *CD11c* | 1.00±0.18 | 0.87±0.11 | 0.5449 |
| *F4/80* | 1.00±0.16 | 1.09±0.07 | 0.6018 |
| *MCP1* | 1.00±0.10 | 1.28±0.17 | 0.4197 |
| *Glut4* | 1.00±0.16 | 0.91±0.08 | 0.6276 |
| *PPARγ* | 1.00±0.12 | 0.78±0.07 | 0.1159 |
| *C/EBPα* | 1.00±0.16 | 0.86±0.08 | 0.2519 |
| *SREBP1* | 1.00±0.12 | 0.83±0.08 | 0.2513 |
| *FAS* | 1.00±0.17 | 0.88±0.11 | 0.5598 |
| *SCD1* | 1.00±0.21 | 0.61±0.07 | 0.0831 |
| *ACC1* | 1.00±0.19 | 0.84±0.13 | 0.4832 |
| *PLIN* | 1.00±0.05 | 0.94±0.04 | 0.3299 |
| *HSL* | 1.00±0.04 | 0.95±0.04 | 0.4322 |

N=8 for each group, * $P<0.05$ Zinc vs Control.

**Table S13** Relative gene expression in the epididymal adipose tissue

| Gene | Control (mean±SEM) | Zinc (mean±SEM) | *P* value |
|---|---|---|---|
| *Leptin* | 1.00±0.14 | 1.64±0.23* | 0.0328 |
| *IL6* | 1.00±0.12 | 1.36±0.11* | 0.0482 |
| *CD11c* | 1.00±0.25 | 1.23±0.28 | 0.5445 |
| *F4/80* | 1.00±0.17 | 1.38±0.16 | 0.1338 |
| *MCP1* | 1.00±0.13 | 0.95±0.07 | 0.7337 |
| *Glut4* | 1.00±0.12 | 0.98±0.05 | 0.8662 |
| *SREBP1* | 1.00±0.16 | 0.89±0.13 | 0.5898 |
| *FAS* | 1.00±0.19 | 0.91±0.08 | 0.6741 |
| *SCD1* | 1.00±0.12 | 1.09±0.06 | 0.5346 |
| *PLIN* | 1.00±0.13 | 0.86±0.05 | 0.3212 |
| *HSL* | 1.00±0.09 | 0.96±0.07 | 0.7488 |

N=8 for each group, * $P<0.05$ Zinc vs Control.

**Table S14** Relative gene expression in 3T3-L1 adipocytes treated with zinc sulfate

| Gene | Control (mean±SEM) | $ZnSO_4$ (mean±SEM) | | | *P* value | | | |
|---|---|---|---|---|---|---|---|---|
| | | | | | post-hoc | | | one-way ANOVA |
| | | 10 μM | 50 μM | 100 μM | 10 μM | 50 μM | 100 μM | |
| *Leptin* | 1.00±0.08 | 0.78±0.20 | 1.52±0.13* | 1.78±0.36 | 0.3567 | 0.0238 | 0.0752 | 0.0271 |
| *IL6* | 1.00±0.07 | 1.51±0.13* | 3.48±0.30* | 2.84±0.09* | 0.0250 | 0.0013 | 0.0001 | <0.0001 |

N=3 for each treatment, * $P<0.05$ Zinc vs Control.

**Table S15** Relative gene expression in 3T3-L1 adipocytes treated with zinc chloride

| | Control (mean±SEM) | $ZnCl_2$ (mean±SEM) | *P* value |
|---|---|---|---|
| *Leptin* | 1.00±0.08 | 1.62±0.21* | 0.0195 |
| *IL6* | 1.00±0.13 | 1.71±0.13* | 0.0030 |

N=3 for each treatment, * $P<0.05$ Zinc vs Control.

**Table S16** Relative protein levels and protein phosphorylation levels in perirenal adipose tissue

| | Control (mean±SEM) | Zinc (mean±SEM) | *P* value |
|---|---|---|---|
| pAKT T308/AKT | 1.00±0.23 | 0.21±0.04* | 0.0040 |
| pAKT S473/AKT | 1.00±0.19 | 1.02±0.27 | 0.9445 |
| PPARγ/Tubulin | 1.00±0.31 | 1.02±0.40 | 0.5472 |
| FAS/Tubulin | 1.00±0.25 | 0.22±0.06* | 0.0006 |
| SCD1/Tubulin | 1.00±0.40 | 0.23±0.12 | 0.0723 |
| pHSL/HSL | 1.00±0.14 | 1.37±0.11 | 0.0560 |

N=8 for each group, * $P<0.05$ Zinc vs Control.

**Table S17** Relative protein levels and protein phosphorylation levels in epididymal adipose tissue

| | Control (mean±SEM) | Zinc (mean±SEM) | *P* value |
|---|---|---|---|
| pAKT T308/AKT | 1.00±0.29 | 1.45±0.37 | 0.2618 |
| pAKT S473/AKT | 1.00±0.26 | 0.93±0.36 | 0.6893 |
| pHSL/HSL | 1.00±0.18 | 0.87±0.15 | 0.5744 |

N=8 for each group, * $P$<0.05 Zinc vs Control.

**Table S18** Relative phosphorylation levels of AKT in 3T3-L1 adipocytes

| | Veh | | Insulin | | Tow-way ANOVA | | | post-hoc $P$ value | | | |
|---|---|---|---|---|---|---|---|---|---|---|---|
| | | | | | | | | $ZnSO_4$ vs Con | | Insulin vs Veh | |
| | Con | ZnSO4 | Con | $ZnSO_4$ | $ZnSO_4$ | Insulin | Interaction | Veh | Insulin | Con | $ZnSO_4$ |
| pAKT T308/AKT | 0.00±0.00 | 5.10±1.03* | 1.00±0.25 | 30.66±3.29* | < 0.0001 | < 0.0001 | 0.0001 | 0.0077 | 0.0008 | 0.0164 | 0.0018 |
| pAKT S473/AKT | 0.00±0.00 | 1.98±0.48* | 1.00±0.12 | 34.60±2.09* | < 0.0001 | < 0.0001 | < 0.0001 | 0.0150 | 0.0001 | 0.0012 | 0.0001 |

N=3 for each treatment, * $P$<0.05 Zinc vs Control.

**Table S19** Serum TAG and FFA levels

| | Control (mean±SEM) | Zinc (mean±SEM) | $P$ value |
|---|---|---|---|
| TAG (mMol/L) | 1.66±0.06 | 1.51±0.08 | 0.1516 |
| FFA (mMol/L) | 1.25±0.05 | 1.17±0.07 | 0.3726 |

N=8 for each group.
